# *In Vitro* and *In Vivo* Anti-tumoral Effects of the Flavonoid Apigenin in Malignant Mesothelioma

**DOI:** 10.3389/fphar.2017.00373

**Published:** 2017-06-19

**Authors:** Laura Masuelli, Monica Benvenuto, Rosanna Mattera, Enrica Di Stefano, Erika Zago, Gloria Taffera, Ilaria Tresoldi, Maria Gabriella Giganti, Giovanni Vanni Frajese, Ginevra Berardi, Andrea Modesti, Roberto Bei

**Affiliations:** ^1^Department of Experimental Medicine, University of Rome “Sapienza”,Rome, Italy; ^2^Department of Clinical Sciences and Translational Medicine, University of Rome “Tor Vergata”,Rome, Italy; ^3^Department of Sports Science, Human and Health, University of Rome “Foro Italico”,Rome, Italy; ^4^Department of Chemistry, University of Rome “Sapienza”,Rome, Italy; ^5^Center for Regenerative Medicine, University of Rome “Tor Vergata”,Rome, Italy

**Keywords:** cancer, mesothelioma, apigenin, apoptosis, polyphenol

## Abstract

Malignant mesothelioma (MM) is a tumor arising from mesothelium. MM patients’ survival is poor. The polyphenol 4′,5,7,-trihydroxyflavone Apigenin (API) is a “multifunctional drug”. Several studies have demonstrated API anti-tumoral effects. However, little is known on the *in vitro* and *in vivo* anti-tumoral effects of API in MM. Thus, we analyzed the *in vitro* effects of API on cell proliferation, cell cycle regulation, pro-survival signaling pathways, apoptosis, and autophagy of human and mouse MM cells. We evaluated the *in vivo* anti-tumor activities of API in mice transplanted with MM #40a cells forming ascites. API inhibited *in vitro* MM cells survival, increased reactive oxygen species intracellular production and induced DNA damage. API activated apoptosis but not autophagy. API-induced apoptosis was sustained by the increase of Bax/Bcl-2 ratio, increase of p53 expression, activation of both caspase 9 and caspase 8, cleavage of PARP-1, and increase of the percentage of cells in subG1 phase. API treatment affected the phosphorylation of ERK1/2, JNK and p38 MAPKs in a cell-type specific manner, inhibited AKT phosphorylation, decreased c-Jun expression and phosphorylation, and inhibited NF-κB nuclear translocation. Intraperitoneal administration of API increased the median survival of C57BL/6 mice intraperitoneally transplanted with #40a cells and reduced the risk of tumor growth. Our findings may have important implications for the design of MM treatment using API.

## Introduction

Malignant mesothelioma (MM) is a tumor arising from mesothelial cells of the serous membranes, most commonly involving the pleural and peritoneal spaces (Antman, 1993). MM are of epithelioid, sarcomatoid, and biphasic types (Inai, 2008). The development of MM has been linked to the exposure to asbestos which causes genotoxic and non-genotoxic damages (Carbone et al., 2012; Izzi et al., 2012a). Abnormal activation of the AP-1/TNF-α/NF-κB autocrine pathway in DNA-damaged mesothelial cells increases their survival and promotes uncontrolled cell growth (Carbone et al., 2012). Mesothelial cells activate inflammation by releasing reactive oxygen species (ROS), nitrogen species (RNS), and cytokines. Inflammatory cells are responsible during the frustrated phagocytosis of asbestos fibers for the free radicals mediated mesothelial cell injury as well (Branchaud et al., 1993; Carbone et al., 2012). In addition, the chronic inflammation and the poor response to therapeutics might be due to the ability of MM cells to subvert host immune response (Branchaud et al., 1993; Kim et al., 2006; Izzi et al., 2009; Chéné et al., 2016). Despite the knowledge of MM carcinogenesis, MM patients’ survival is poor, and it was slowly improving in the last decades (Howlader et al., 2009). The therapeutic strategies for the treatment of malignant pleural mesothelioma are referred to as ‘life-extending treatments’ (Favoni and Florio, 2011). Intra serous sac administration of drugs in the peritoneal or pleural space might improve the treatment of MM avoiding or diminishing therapy side effects and increasing drugs’ bioavailability. Taking into account the poor outcome and toxicity of chemotherapy, novel approaches based on targeting abnormal activated signaling pathways in MM cells were employed to improve MM patients survival. Preclinical trials have used a second generation of drugs including inhibitors of mTOR, folate, receptor tyrosine kinase and ciclooxygenase, while clinical trials have been carried out using chemotherapy with proteasome, mTOR and histone deacetylases inhibitors (Favoni and Florio, 2011). However, although single targeted therapies have shown to ameliorate the quality of patients life and their survival and the use of new generation of folate inhibitors alone or in combination with platinum derivatives showed promising results, the patients absolute response rate was insufficient as compared to other tumors (Favoni and Florio, 2011).

4′,5,7,-trihydroxyflavone, commonly called Apigenin, is a flavonoid contained in fruits and vegetables such as orange, grapefruit, onion, parsley, basil, celery, tea leaf, licorice root, wheat sprouts (Shukla and Gupta, 2010; Izzi et al., 2012b; Benvenuto et al., 2016c; Grosso et al., 2016; Sung et al., 2016; Ganai, 2017). Apigenin has been demonstrated to have anti-tumoral effects due to its ability to inhibit the growth of a variety of human cancer cell lines *in vitro* (Shukla and Gupta, 2010; Masuelli et al., 2011; Bao et al., 2013; Tong and Pelling, 2013; Chen et al., 2014; Lee et al., 2014; Wu et al., 2014; Liu et al., 2015; Seo et al., 2015; Shi et al., 2015; Shukla et al., 2015; Kim et al., 2016; Sung et al., 2016; Xu et al., 2016; Lim et al., 2016; Ganai, 2017). Apigenin induces a G0/G1 and G2/M cell cycle arrest through suppression of cyclin B-associated cdc2 activity and phosphorylation of Rb, induction of p21 and p27 and down-regulation of cyclin D1, D3, and cdk4 (Lepley and Pelling, 1997; Yin et al., 2001; Ujiki et al., 2006; Shukla and Gupta, 2007; Hussain et al., 2010). Apigenin was reported to activate both the intrinsic and extrinsic apoptotic pathways in cancer cells (Chen et al., 2014; Lee et al., 2014; Seo et al., 2015; Shi et al., 2015; Sung et al., 2016) and in few experimental models to induce simultaneous autophagy (Sung et al., 2016). Several signaling pathways were shown to be inhibited by apigenin in cancer cells (Lepley and Pelling, 1997; Yin et al., 2001; Ujiki et al., 2006; Shukla and Gupta, 2007; Hussain et al., 2010; Shukla and Gupta, 2010; Masuelli et al., 2011; Bao et al., 2013; Tong and Pelling, 2013; Chen et al., 2014; Lee et al., 2014; Wu et al., 2014; Liu et al., 2015; Seo et al., 2015; Shi et al., 2015; Shukla et al., 2015; Sung et al., 2016; Kim et al., 2016; Lim et al., 2016; Xu et al., 2016; Ganai, 2017). Apigenin was able to inhibit the phosphorylation of EGFR, ErbB2, and mitogen activated protein (MAP) kinase and the activity of PI3K/AKT (Masuelli et al., 2011; Lim et al., 2016). Apigenin has also been shown to limit cancer cells invasion by inhibiting FAK/Src signaling and tumor angiogenesis (Fang et al., 2007; Franzen et al., 2009). Apigenin limited the activation of the Wnt/β-catenin signaling pathway (Liu et al., 2015; Xu et al., 2016), and the activity of NF-κB (Wu et al., 2014; Shukla et al., 2015). In addition, apigenin has been shown to block the phosphorylation of c-Met and its downstream effectors (Kim et al., 2016).

To our knowledge no studies were performed to analyze the effect of apigenin on signal transduction pathways activated in MM cells and on the *in vivo* growth of MM cells. Thus, in this report we evaluated for the first time the *in vivo* effect of intratumoral administration of API in a mouse model in which MM cells form ascites after transplantation in the peritoneal cavity. In addition, we evaluated *in vitro* effects of API on cell growth, cell cycle regulation, pro-survival signaling pathways, apoptosis and autophagy in human and mouse MM cell lines.

## Materials and Methods

### Reagents

DMSO, 4′,5,7,-trihydroxyflavone (Apigenin, API), Sulforhodamine B (SRB), Hoechst 33342 and DAPI were purchased from Sigma–Aldrich (Milano, Italy). Antibodies against AKT, phospho-AKT, p38 and phospho-p38, JNK and phospho-JNK, caspase 9, caspase 8, c-Jun, phospho-c-Jun, IκBα, and phospho-IκBα were obtained from Cell Signaling Technology (Boston, MA, United States). Antibodies against Bax, Bcl-2, and γ-H2AX were obtained from BD Pharmigen (BD Biosciences, San Jose, CA, United States). Antibodies against p53, PARP-1, ERK1/2 (C-14), phospho-ERK (E-4), NF-κB (p65) were obtained from Santa Cruz Biotechnology (Santa Cruz, CA, United States). Antibodies against Beclin-1 and p62/SQSTM1 were obtained from Abcam (Cambridge, United Kingdom). The anti-LC3 antibody was purchased from Novus Biologicals (Littleton, CO, United States). Peptide antisera to human EGFR and ErbB2 receptors have previously been characterized for detection specificity by immunohistochemistry and immunoblotting (Fedi et al., 1994; Alimandi et al., 1995; Bei et al., 1999).

Goat anti-mouse IgG Alexa fluor-488-conjugated secondary antibody was purchased from Invitrogen (Milano, Italy). The rabbit polyclonal antibodies against actin and tubulin and goat anti-mouse or the anti-rabbit IgG peroxidase-conjugated secondary antibodies were obtained from Sigma-Aldrich.

### Cell Lines and Treatments

Human MM cell lines [MM-F1 (fibromatous), MM-B1 (biphasic), and H-Meso-1 (epithelioid)] and the murine MM cell line #40a were maintained in DMEM (*Dulbecco’s modified Eagle’s medium*) containing 10% fetal bovine serum, 100 U/ml penicillin and 100 μg/ml streptomycin (complete medium). The cells were grown at 37°C in a humidified incubator with an atmosphere of 5% CO_2_. Isolation of the murine mesothelioma 40-cell line was previously described by Goodglick et al., 1997. The #40a cell line is derived from the 40-cell line after two passages in the peritoneal cavity of C57BL/6 mice following administration of pristane one week before cells transplant. These passages allow the selection of cells which reproducibly form ascites when intraperitoneally injected in the mice. H-Meso-1 cells have an epithelial morphology, while MM-B1 and MM-F1 cells have biphasic and sarcomatous features, respectively (Palumbo et al., 2008). The 40-cell line has an epithelial morphology (Goodglick et al., 1997). API was dissolved in DMSO. For the treatments, the cells were incubated for the indicated times in the presence of API (dose range: 6.25–100 μM) or the vehicle (DMSO ≤ 0.1%).

### Sulforhodamine B (SRB) Assay

Cells were seeded at 5 × 10^3^/well in 96-well plates and incubated at 37°C to allow cell attachment. After 24 h, the medium was changed and the cells were treated with API or DMSO and incubated for 24 h, 48 h, 72 h at concentrations of 6.25–12.5–25–50–100 μM. The cells were then fixed with cold trichloroacetic acid (final concentration 10%) for 1 hour at 4°C. After four washes with distilled water, the plates were air-dried and stained for 30 min with 0.4% (wt/vol) SRB in 1% acetic acid. After four washes with 1% acetic acid to remove the unbound dye, the plates were air-dried, and cell-bound SRB was dissolved with 200 μl/well of 10 mM unbuffered Tris base solution. The optical density (O.D.) of the samples was determined at 540 nm with a spectrophotometric plate reader. The percentage survival of the cultures treated with API was calculated by normalizing their O.D. values to those of control cultures treated with DMSO (Benvenuto et al., 2016b; Masuelli et al., 2017). The experiments were performed in triplicate and repeated three times.

### Trypan Blue Exclusion Test

For trypan blue exclusion test, cells were seeded at 5 × 10^4^/well in 24-well plates and incubated at 37°C to allow cells attachment. After 24 h, the medium was changed and the cells were treated with API or DMSO and incubated for 24 h, 48 h, 72 h at concentrations of 6.25–12.5–25–50–100 μM. After 24 h, 48 h, and 72 h, adherent as well as suspended cells of each well were harvested and stained with trypan blue (Sigma–Aldrich, Milan, Italy) and counted with an optic microscope (Strober, 2001). The experiments were repeated three times. Percentage of cells death was determined compared to the total number of cells (Benvenuto et al., 2015).

### Fluorescent Measurement of ROS

Dichlorofluorescin diacetate (DCF-DA) was used to detect ROS production in cells. Briefly, 2.5 × 10^5^ cells were seeded into 6-well plates and incubated at 37°C to allow cell attachment before treatment. After two washings with PBS, cells were incubated with 10 μM 2’,7’-dichlorofluorescein diacetate (Sigma–Aldrich, Milan, Italy) in PBS at 37°C and 5% CO_2_ in the dark for 30 min (Masuelli et al., 2016). After two washings, cells were treated with API (6.25–100 μM) or DMSO in serum-free medium and incubated at 37°C and 5% CO_2_ in the dark for different times (15 min-4 h). Then, adherent cells and suspended cells were harvested, centrifuged at 1250 rpm for 10 min, and seeded in 96-well plates (100 μl per well). Fluorescence intensity was measured after 15 and 30 min and 1 and 4 h using a spectrophotometric plate reader at an excitation wavelength of 495 nm and an emission wavelength of 535 nm. Because the highest level of fluorescence was detected at 30 min and then decreased back to the level of the control after 1 h of stimulation (data not shown), this experimental time was chosen for subsequent experiments.

### FACS Analysis

Asynchronized, log-phase growing cells (60% confluent, approximately 2.5 × 10^5^/well in 6-well plates) were treated with API (6.25–12.5–25–50–100 μM) or DMSO in complete culture medium. After 48 h adherent as well as suspended cells were harvested, centrifuged at 1500 rpm for 10 min and washed twice with cold phosphate-buffered saline (PBS). The cell pellets were re-suspended in 70% ethanol and incubated for 1 h at –20°C. The cells were then washed twice with cold PBS, centrifuged at 1500 rpm for 10 min, incubated for 1 h in the dark with propidium iodide (25 μg/ml final concentration in 0.1% citrate and 0.1% Triton X-100) and analyzed by flow cytometry using a FACSCalibur cytometer with CellQuest software (Masuelli et al., 2014).

### Preparation of Cell Lysates and Western Blotting

Approximately 1 × 10^6^ cells were seeded in 100-mm tissue culture dishes 24 h prior to the addition of 50 μM API or vehicle. After 24 h of incubation, the cells were harvested, washed twice with cold PBS and lysed in RIPA lysis buffer (Triton X-100 1%, SDS 0.1%, NaCl 200 mM, Tris HCl 50 mM pH 7.5, PMSF 1 mM, and NaOV 1 mM). After 30 min at 4°C, the mixtures were centrifuged at 12000 *g* for 15 min and the supernatants were analyzed by Western blotting. For Western blotting analysis, 50 μg of cell lysates were resolved in 10% SDS-PAGE and then transferred to nitrocellulose membranes. After blocking, the membranes were incubated with specific primary antibodies at 1–2 μg/ml concentrations overnight at 4°C. After being washed, the filters were incubated with goat anti-mouse or anti-rabbit IgG, peroxidase-conjugated antibodies and developed by chemiluminescence as previously described (Masuelli et al., 2017). A densitometric analysis of autoradiographic bands was performed with Image J software (National Institutes of Health, United States) after blot scanning.

### Immunofluorescence

Cells were seeded at 4 × 10^4^ cells/well in 8-well chamber slides and, after 24 h, they were treated with 50 μM API, or with the vehicle. After 24 h, the cells were fixed in 4% formaldehyde for 10 min, washed and fixed in methanol for 5 min at –20°C, then washed again and incubated with specific primary antibody against NF-κB for 1 h at room temperature. After additional washings, the cells were labeled with a goat anti-mouse IgG Alexa fluor-488-conjugated secondary antibody for 30 min (Masuelli et al., 2012). After a third washing, the cells were incubated with 0.1 μg/ml DAPI and mounted under a cover slip with glycerol. The cells were observed with an Olympus BX51 microscope.

### *In Vivo* Treatment of C57BL/6 Mice Intraperitoneally Administered with API and Transplanted with MM #40a Cells

Groups of 6-to-8-weeks-old C57BL/6 mice (6 mice per group) were intraperitoneally (i.p.) inoculated with 0.2 ml of suspension containing 1 × 10^6^ #40a cells in PBS 1 week after pristane injection (250 μl). Mice were treated i.p. weekly with 20 mg/kg API (dissolved in DMSO and then diluted in 400 μl PBS). The control group was injected weekly with the same amount of vehicle solution (DMSO-PBS). The treatments were started simultaneously with the inoculation of cells.

Investigation has been conducted in accordance with the ethical standards and according to the Declaration of Helsinki. A veterinary surgeon was present during the experiments. The animal care both before and after the experiments was performed only by trained personnel. Mice were bred under pathogen-free conditions in the animal facilities of the University of Rome “Tor Vergata” and handled in compliance with European Union and institutional standards for animal research. The work was conducted with the formal approval of the local [“Organismo Preposto al Benessere degli animali” (O.P.B.A.), University of Rome Tor Vergata, http://www.sta.uniroma2.it] and national (Ministry of Health) animal care committees and animal experiments have been registered as legislation requires (Authorization from Ministry of Health n° 187/2016-PR).

### Analysis of Antitumor Activity *In vivo*

MM #40a cells growth in the peritoneum forms ascites. Accordingly, the abdominal circumference of mice was monitored before the inoculation of cells and every week until tumor-bearing mice were euthanized at the first signs of distress or when their abdominal circumference exceeded 12 cm (Masuelli et al., 2017).

### Statistical Analysis

The data distribution of cell survival, cell death, ROS production and the FACS analyses were preliminarily verified by Kolmogorov–Smirnov test, and data sets were analyzed by one-way analysis of variance (ANOVA) followed by Newman-Keuls test. Differences in the intensity of immunoreactive bands were evaluated by a two-tailed Student’s *t*-test. Values with *p* ≤ 0.05 were considered significant. Survival curves and tumor volumes were estimated using the Kaplan–Meier method and compared with a log-rank test (Mantel-Cox). Differences in tumor volumes were regarded as significant when the *p*-value was ≤0.05 (Benvenuto et al., 2016a; Masuelli et al., 2017).

## Results

### Cytotoxicity of API on MM Cell Lines

**FIGURE 1:**
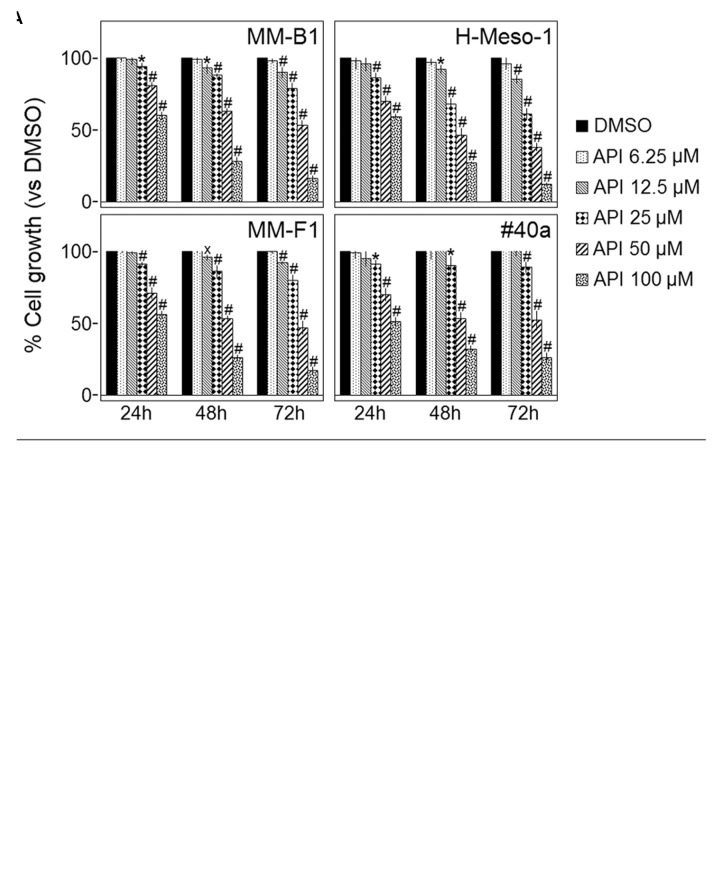
Effect of Apigenin (API) on malignant mesothelioma (MM) cell lines growth and death. **(A)** The growth of human (MM-B1, H-Meso-1, MM-F1) and mouse (#40a) MM cell lines were assessed by the SRB assay after 24, 48, and 72 h of treatment with DMSO or API. The percentage of cells growth treated with API was calculated by normalizing the O.D. value to that of the control cultures (DMSO). The results are expressed as the mean ± SD of three independent experiments performed in triplicate (^x^*p* ≤ 0.05, ^∗^*p* ≤ 0.01, #*p* ≤ 0.001 compared with the cultures treated with DMSO). **(B)** Trypan blue exclusion test was performed to determine the percentage of cell death of MM cells treated with API or DMSO after 24, 48, and 72 h of treatment. The results are expressed as the mean ± SD of three independent experiments performed in triplicate (^x^*p* ≤ 0.05, ^∗^*p* ≤ 0.01, #*p* ≤ 0.001 compared with the cultures treated with DMSO).

Cell growth of human MM cell lines with different histotypes [MM-B1 (biphasic), H-Meso-1 (epithelioid), and MM-F1 (fibromatous)] and of a mouse (#40a) MM cell line was quantified after exposure to increasing doses of Apigenin (API) (6.25 to 100 μM) or vehicle control for 24, 48, and 72 h by SRB and Trypan blue exclusion assays. The effect of API was dose- and time-dependent and reached statistical significance at the doses 12.5, 25, 50, and 100 μM after 48 and 72 h in all human cells and at the doses of 25, 50, and 100 μM in #40a cells after 24–72 h of exposure (**Figure 1A**).

The dye exclusion test was used to determine the number of viable MM cells upon API exposure. The cytotoxic effect of API was dose- and time-dependent. The percentage of cell death upon API treatment was 82, 28 e 9 for MM-B1 (*p* < 0.001); 78, 41 e 19 for MM-F1 (*p* < 0.001); 83, 48 e 24 for H-Meso-1 (*p* < 0.001) cells and 69, 42 e 18 for #40a (*p* < 0.001) cells after 72 h of exposure at the highest doses (**Figure 1B**).

The concentrations of API required to reduce cell growth by 50% (IC50) were 49.16 ± 2.52 μM, 46.95 ± 1.69, and 34.31 ± 1.55 μM for MM-B1, MM-F1, and H-Meso-1 after 72 h, respectively and 56.82 ± 4.69 for #40a after the same time of exposure (**Table 1**).

**Table 1:** Apigenin (API) concentrations required for 50% inhibition of malignant mesothelioma (MM) cell lines growth (IC50).

MM cell lines	API treatment (hours)	IC50 ± SD (μM)
**MM-B1**	48	64.23 ± 2.73
	72	49.16 ± 2.52
**MM-F1**	48	56.31 ± 2.13
	72	46.95 ± 1.69
**H-Meso-1**	48	46.44 ± 4.08
	72	34.31 ± 1.55
**#40a**	48	60.39 ± 3.62
	72	56.82 ± 4.69

### API Induces ROS Generation in MM Cells and DNA Damage

**FIGURE 2:**
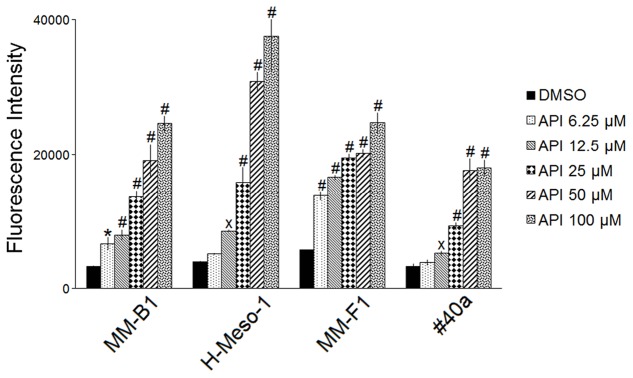
Effect of API on the intracellular ROS production in MM cell lines. Results are reported as the mean of the fluorescence intensity ± SD values from three experiments performed in triplicate. API was used in the range 6.25–100 μM. Statistical significance of the effects obtained with API was calculated *vs.* those obtained with DMSO (^x^*p* ≤ 0.05, ^∗^*p* ≤ 0.01, #*p* ≤ 0.001).

It was reported that API increases ROS in cancer cells (Wang and Zhao, 2017). To determine the effect of API on intracellular ROS production, the DCF-DA assay was performed in API-treated MM cells. The effect of the compound was compared to that of DMSO and the results were expressed as the mean of the fluorescence intensity (**Figure 2**). API induced a significant dose-dependent ROS production as compared to the vehicle in all MM cells. API induced a significant ROS production even at the lower dose in MM-B1 and MM-F1 cells.

**FIGURE 3:**
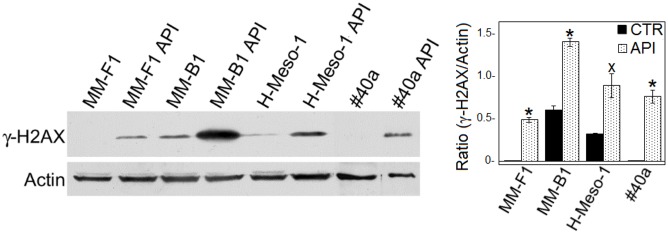
Effect of API on DNA damage in MM cells. The expression of γ-H2AX was determined by Western blotting analysis in MM cell lines treated with API at 50 μM or with DMSO (CTR) for 24 h. Actin was used as an internal control. The intensity of the bands was quantified using the ImageJ software after blot scanning of two independent experiments. The densitometric ratios between γ-H2AX and actin and statistical analysis are reported. Data are expressed as the mean ± SD of two independent experiments (^x^*p* ≤ 0.05, ^∗^*p* ≤ 0.01 compared with CTR).

ROS cause DNA damage that rapidly results in the phosphorylation of the histone H2A variant (H2AX) at Ser 139 (γ-H2AX) (Sharma et al., 2012). Treatment with API at the concentration of 50 μM for 24 h led to a significant increased of γ-H2AX in all MM cell lines (MM-F1, *p* = 0.001; MM-B1, *p* = 0.003; H-Meso-1, *p* = 0.03; #40a, *p* = 0.005) (**Figure 3**). The higher increase of H2AX phosphorylation observed in MM-B1 cells might reflect the behavior of its histotype or the distinctive expression of DNA repair enzymes in this cell line.

### API Activates Apoptosis But Not Autophagy in MM Cells

**FIGURE 4:**
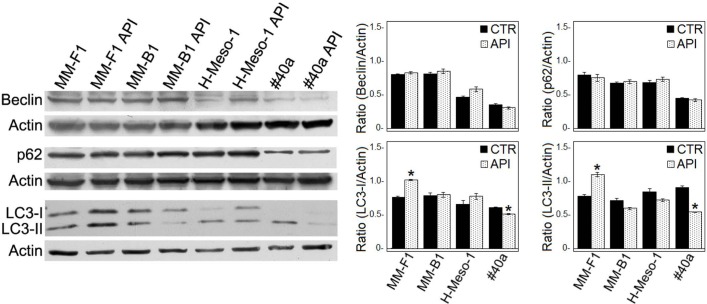
Effect of API on the autophagic flux in MM cells. The expression of Beclin-1, p62/SQSTM (p62), and LC3-I and LC3-II was assessed by Western blotting in MM cell lines treated with API at 50 μM or DMSO (CTR) for 24 h. Actin was used as an internal control. The intensity of the bands obtained was quantified using the ImageJ software after blot scanning of two independent experiments. The densitometric ratios between Beclin-1 and actin, LC3-I and actin, LC3-II and actin, p62 and actin and statistical analysis are reported. Data are expressed as the mean ± SD of two independent experiments (^∗^*p* ≤ 0.01 compared with CTR).

Autophagy represents the ability of the cell to adapt to stress. In order to demonstrate whether API treatment was able to induce autophagy in MM cell lines, the expression of proteins involved in autophagy (i.e., Beclin-1, p62/SQSTM, and LC3) was investigated by Western blotting in API-treated MM cell lines. As shown in **Figure 4**, the expression levels of Beclin-1 and p62 remained unchanged in all MM cell lines. During autophagy, cytosolic LC3-I is modified to a membrane-bound form (LC3-II) that localizes to autophagosomes, making this protein an autophagosomal marker (Kabeya et al., 2000). LC3-I and/or LC3-II were constitutively expressed in DMSO-treated MM cell lines. API treatment induced a slight significant increase of LC3-II only in MM-F1 cells while a significant decrease in LC3-II has been shown in #40a cells. These results suggest that API does not induce autophagy in MM cell lines (**Figure 4**).

**FIGURE 5:**
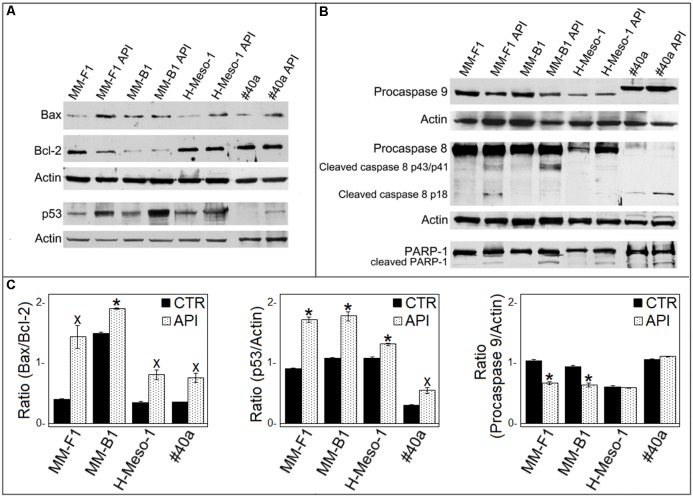
Effect of API on the expression of molecules involved in apoptosis in MM cells. **(A)** The expression of Bax, Bcl-2, p53 was assessed by Western blotting analysis in MM cells treated for 24 h with API at 50 μM or with DMSO (CTR) as vehicle. Actin was used as an internal control. **(B)** The expression of procaspase 9, procaspase 8, cleaved caspase 8 fragments, and the cleavage of PARP-1 were assessed by Western blotting analysis in MM cells treated for 24 h with API at 50 μM or with DMSO (CTR) as vehicle. Actin was used as an internal control. The intensity of the bands was quantified using ImageJ software after blot scanning, obtained from two independent experiments. **(C)** The densitometric ratios between Bax and Bcl-2, between p53 and actin, and between procaspase 9 and actin and statistical analysis are reported. Data are expressed as the mean ± SD of two independent experiments (^x^*p* ≤ 0.05, ^∗^*p* ≤ 0.01 compared with CTR).

It was reported that when the stress is too potent, the process of autophagy is circumvented and the apoptosis is activated (Mariño et al., 2014). The role of p53 in repressing autophagy and activating multiple pro-apoptotic genes prompted us to evaluate p53, Bax, and Bcl-2 expression in MM cells upon API (50 μM for 24 hours) treatment by Western blotting. Bax expression was strongly increased in API-treated MM-F1, H-Meso-1 and #40a cells, while the increase was moderate in MM-B1 cells. In addition, the expression of Bcl-2 was down-regulated in MM-F1-treated cells compared to DMSO-treated cells. Thus, Bax/Bcl-2 ratio increased in MM cell lines treated with API as compared to cells treated with DMSO (MM-F1, *p* = 0.017; MM-B1, *p* = 0.02; H-Meso-1, *p* = 0.014; #40a, *p* = 0.018) (**Figures 5A,C**).

Furthermore, API increased p53 expression compared to DMSO treatment in MM-F1 (*p* = 0.001), MM-B1 (*p* = 0.0063), H-Meso-1 (*p* = 0.008), and #40a (*p* = 0.02) cells (**Figures 5A,C**). The activation of the intrinsic pathway of the apoptosis is sustained by the activation of the procaspase 9 into caspase 9 or decrease of procaspase 9 (Wang and Zhao, 2017). API was able to decrease procaspase 9 expression in MM-F1 and MM-B1 cells as compared to DMSO treated cells (*p* = 0.0033 and 0.0093, respectively) (**Figures 5B,C**). The levels of procaspase 9 remained unchanged in H-Meso-1 and #40a cells.

Next, we determined whether API was able to activate apoptosis through the extrinsic pathway as well. API activated procaspase 8 as detected by Western blotting analysis showing the presence of caspase 8 cleavage fragments (p43/41 and/or p18) in all treated MM cells (**Figure 5B**).

The cleavage of poly (ADP-ribose) polymerase-1 (PARP-1) impairs DNA repairs and genomic integrity sustaining apoptosis, and is mainly mediated by activation of caspase 3 (Kaufmann et al., 1993). Thus, API-mediated cleavage of PARP-1 was analyzed by Western blotting. API treatment induced PARP-1 proteolytic cleavage in all MM cells (**Figure 5B**).

**FIGURE 6:**
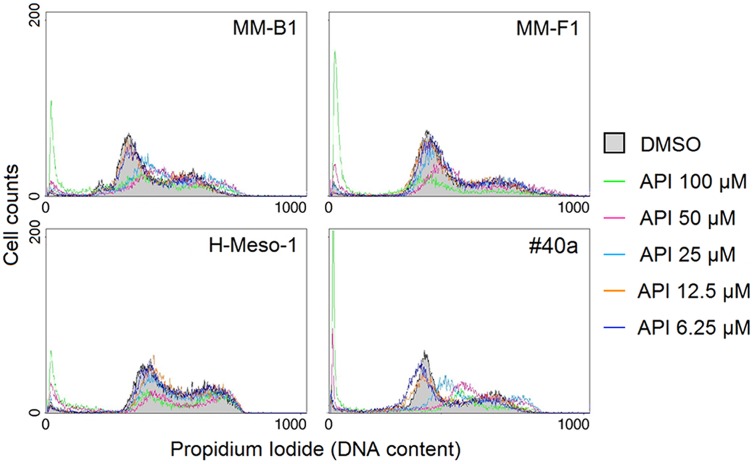
Effect of API on cell cycle distribution. FACS analysis of DNA content was performed on asynchronized log phase growing MM cells treated for 48 hours with DMSO or API at 6.25–100 μM. A representative experiment is shown in the figure.

**FIGURE 7:**
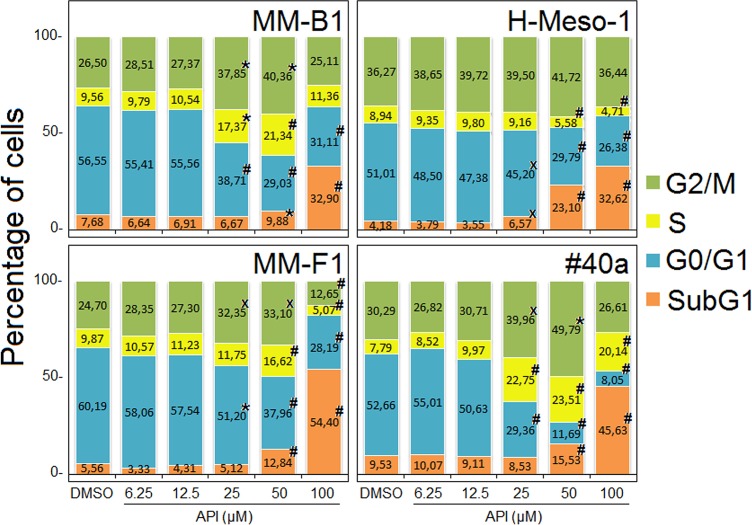
Stacked bar graphs showing the percentage of cells in different phases of the cell cycle. Percentage of cells in subG1, G0/G1, S, and G2/M phases was calculated with CellQuest Pro 5.2 software. Results represent mean values from three independent experiments. Statistical significance of the effects obtained with API treatment was calculated vs. those obtained in DMSO-treated cells with one-way ANOVA analysis of variance (^x^*p* ≤ 0.05, ^∗^*p* ≤ 0.01, #*p* ≤ 0.001).

To further evaluate the effect of API on apoptosis, the analysis of the cell cycle distribution of API-treated MM cells was performed by FACS analysis of DNA content. **Figure 6**, shows a representative experiment in which the effect of increasing doses of API on DNA content were compared to that obtained with DMSO. The mean results of three independent experiments are reported in **Figure 7**. Our results demonstrated that API induced a dose-dependent increase in the percentage of cells in subG1 in all MM cell lines. The hypodiploid DNA content detected in the subG1 phase is typical of apoptosis. Such effect was associated with a decrease of the percentage of cells in G0/G1 in all MM cell lines and with an increase of the percentage of cells in G2/M in MM-B1, MM-F1, and #40a cells.

### API Decreases EGFR and ErbB2 Expression, Increases the Phosphorylation of ERK1/ERK2 and p38 But Diminishes that of c-Jun and AKT

**FIGURE 8:**
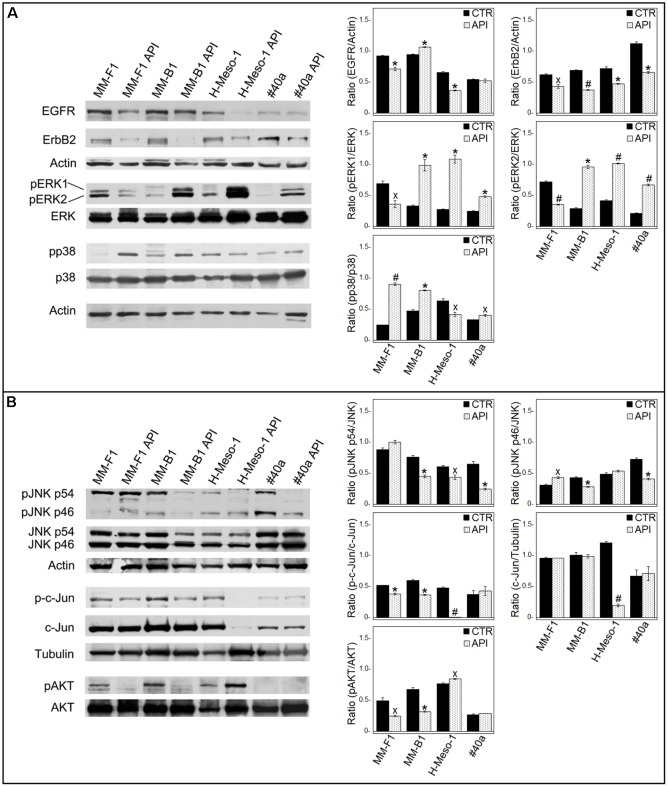
Effect of API on the expression and activation of signaling pathway molecules. Western blotting analysis was performed on MM cells treated with API (50 μM) or DMSO (CTR) for 24 h. **(A)** The levels of pERK1 and pERK2 proteins, as well as p-p38 protein were compared with that of total ERK and p38 proteins, respectively. **(B)** The levels of pJNK p54, pJNK p46, p-c-Jun, and pAKT were compared with that of total JNK, c-Jun and AKT proteins, respectively. The ratios and statistical analysis are reported. Data are expressed as the mean ± SD of two independent experiments (^x^*p* ≤ 0.05, ^∗^*p* ≤ 0.01, #*p* ≤ 0.001 compared with CTR). Actin and tubulin were used as an internal control.

ErbB receptors signaling transduction pathway is activated in mesothelial cells by asbestos (Heintz et al., 2010). Thus, the expression of EGFR and ErbB2, the expression and phosphorylation of mitogen-activated protein (MAP) kinases including ERK1/2, the p38 kinase and the c-Jun N-terminal kinases (JNKs p54 and p46) were analyzed by Western blotting after API cells treatment (**Figure 8**).

The levels of phosphorylated proteins were compared with the total proteins level. As shown in **Figure 8A**, API treatment was able to reduce the expression of EGFR in human MM cells (MM-F1, *p* = 0.0091; MM-B1, *p* = 0.0035; H-Meso-1, *p* = 0.0014) and of ErbB2 in human and mouse MM cell lines (MM-F1, *p* = 0.013; MM-B1, *p* = 0.0005; H-Meso-1, *p* = 0.007; #40a, *p* = 0.0024). In addition, treatment with API increased the level of phosphorylation of ERK1 and ERK2 in MM-B1 (*p* = 0.009 and *p* = 0.001, respectively), H-Meso-1 (*p* = 0.003 and *p* = 0.0003, respectively), and #40a (*p* = 0.0022 and *p* = 0.0006, respectively) cells compared to DMSO-treated cells. Conversely, the effect was opposite in MM-F1-treated cells (*p* = 0.022 and *p* = 0.0009, respectively). p38 phosphorylation was increased upon API treatment in MM-F1 (*p* = 0.0005), MM-B1 (*p* = 0.002), and #40a (*p* = 0.0198) cells. On the other hand, p38 phosphorylation was decreased in H-Meso-1 (*p* = 0.024) cells (**Figure 8A**). p54 JNK phosphorylation was significantly diminished in MM-B1 and H-Meso-1 cells (*p* = 0.0067 and *p* = 0.02, respectively) and it was abolished in #40a cells (*p* = 0.006) compared to control-treated cells. p54 JNK phosphorylation was unchanged in MM-B1 cells. p46 JNK phosphorylation was decreased in MM-B1 and #40a cells (*p* = 0.005 and *p* = 0.0033, respectively), while it was increased in MM-B1 (*p* = 0.0136) and remained unchanged in H-Meso-1 cells, compared to control-treated cells (**Figure 8B**).

Apigenin treatment significantly decreased the expression of c-Jun in H-Meso-1 as compared to DMSO-treated cells (*p* = 0.0004). Of note, a decreased c-Jun phosphorylation was observed in MM-F1 (*p* = 0.005), MM-B1 (*p* = 0.002), and H-Meso-1 (*p* = 0.0004) upon API cells treatment (**Figure 8B**). c-Jun phosphorylation was not affected by API cells treatment in #40a cells.

Finally, API cells treatment abolished AKT phosphorylation in MM-F1 (*p* = 0.0192) and MM-B1 (*p* = 0.0031) cells while increased it in H-Meso-1 (*p* = 0.012) cells. p-AKT was not detected in #40a cells (**Figure 8B**).

### API Inhibits NF-κB Nuclear Translocation

**FIGURE 9:**
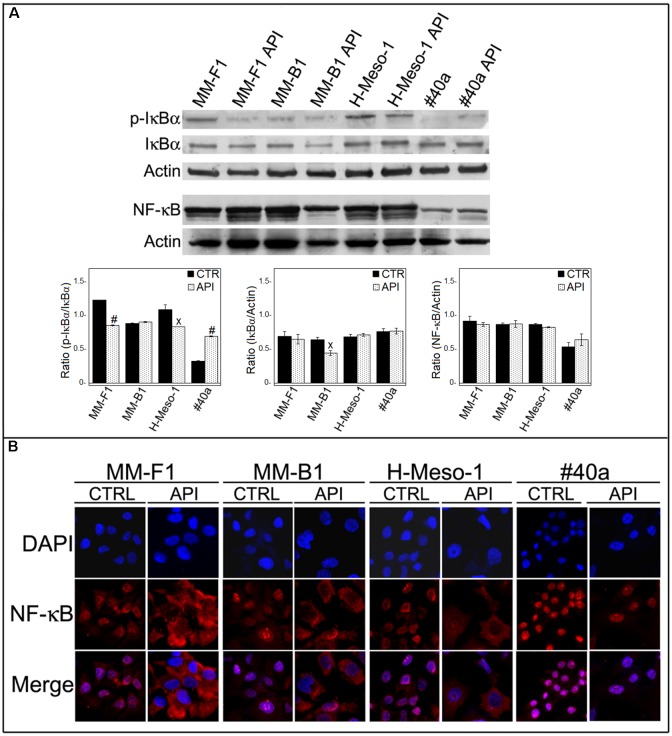
Effect of API on NF-κB activation. **(A)** Western blotting analysis was performed on MM cells treated with API at 50 μM or with the DMSO vehicle (CTR) for 24 h. The densitometric ratios between NF-κB and actin, IκBα and actin, p-IκBα and IκBα and statistical analysis are reported. Data are expressed as the mean ± SD of two independent experiments (^x^*p* ≤ 0.05, #*p* ≤ 0.001 compared with CTR). **(B)** Inhibition of nuclear translocation of NF-κB after treatment with API in MM cells was assessed by immunofluorescence analysis. Cells were fixed after treatment, and incubated with the anti-NF-κB antibody. After two washes with PBS, the cells were incubated with the goat anti-mouse IgG Alexa fluor-488-conjugated secondary antibody. Nuclei were stained with DAPI. Original magnification x400.

Apigenin has been demonstrated to inhibit NF-κB both *in vitro* and *in vivo* in cancer cells (Wu et al., 2014; Shukla et al., 2015). No results are available on the effect of API on NF-κB in MM cells. Thus, we determined whether API was able to modulate NF-κB expression and/or activation in MM cells. Treatment with API did not affect the expression of NF-κB in MM cells as observed by Western blotting (**Figure 9A**).

NF-κB acts as a transcription factor, moving into its active form in the nucleus. The activation of NF-κB is regulated by phosphorylation and degradation of IκBα. Unphosphorylated IκBα blocks NF-κB in the cytoplasm while phosphorylated IκBα is normally ubiquitinated and degraded leaving the active NF-κB which is then able to translocate into the nucleus. To determine whether API treatment was able to affect NF-κB nuclear translocation, we analyzed the expression and phosphorylation of IκBα by Western blotting and NF-κB localization by immunofluorescence analysis. Phosphorylation of IκBα was significantly decreased in API-treated MM-F1 and H-Meso-1 cells compared to DMSO-treated cells (*p* = 0.00018 and *p* = 0.035, respectively) while it was increased in #40a cells upon API treatment (*p* = 0.0004) (**Figure 9A**). In addition, NF-κB was found to be mainly localized in the nucleus in DMSO-treated MM cells. Conversely, API treatment induced the accumulation of NF-κB in the cytoplasm of MM-F1, MM-B1 and H-Meso-1 cells, but not in #40a cells. This result indicates an inhibitory effect of API on NF-κB nuclear translocation in human MM cell lines (**Figure 9B**).

### API Reduces Tumor Growth in C57BL/6 Mice Intraperitoneally Transplanted with MM #40a Cells

**FIGURE 10:**
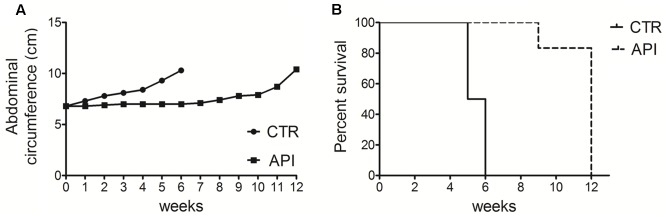
Apigenin reduced tumor growth and increased the survival in C57BL/6 mice intraperitoneally transplanted with MM #40a cells. **(A)** Differences in mean abdominal circumferences between C57BL/6 mice treated with API or with DMSO-PBS (CTR). **(B)** Differences in the mean survival duration of C57BL/6 mice treated with API or with DMSO-PBS (CTR). The numbers of inoculated mice are reported in the Section “Materials and Methods”.

To evaluate the *in vivo* antitumor effects of API, C57BL/6 mice (6 mice per group) were intraperitoneally inoculated with 1x10^6^ syngeneic MM cells (#40a). Mice were simultaneously intraperitoneally administered with 20 mg/kg API dissolved in DMSO-PBS or with the vehicle alone (CTR). The treatment was performed weekly. Since #40a cells form ascites, the measurement of the abdominal circumference of the mice was assessed prior to cells inoculation and then every week. After 2 weeks of treatment, mice treated with API showed a significant lower abdominal circumference than control mice (mean value 6.9 cm compared with 7.8 cm, *p* = 0.0072) (**Figure 10A**).

All control mice were euthanized at 5 (three mice) and 6 (three mice) weeks of treatment, for the excessive size of their tumors. Conversely, all API-treated mice remained alive at this time (mean abdominal circumference 7.0 cm). API-treated mice were euthanized at 9 weeks (mouse mouse) and at 12 weeks (five mice) of treatment.

Overall, when comparing the survival of C57BL/6 mice upon treatment, it was observed that API treatment prolonged mice median survival time as compared to the vehicle treatment (12 vs. 5.5 weeks; *p* = 0.0009) (**Figure 10B**). The risk of tumor growth in the vehicle-treated mice was 23.17 relative to API-treated mice (**Table 2**). Our results indicated that API specifically interfered with intraperitoneally transplanted MM #40a cells growth.

**Table 2:** Analysis of the survival of C57BL/6 mice after treatment with API by the log-rank test (Mantel-Cox).

Variable	Contrast	Hazard Ratio	95%Hazard Ratio Confidence Limits		Median Survival (weeks)	p Value
			Lower	Upper		
Treatment	CTR vs API	23.17	3.617	148.4	5.5 vs 12	0.0009

*CTR = control; API = apigenin.*

## Discussion

The survival of MM patients remains poor although multimodality-approaches including surgery, chemotherapy and radiation have increased the quality of patient’s life (Favoni and Florio, 2011). To date Clinicaltrials.gov listed more than one hundred registered trials worldwide evaluating multiple therapeutic approaches for MM treatment (ClinicalTrials.gov, 2017). A large number of new drugs have been evaluated in preclinical studies and early clinical trials in MM patients (Kotova et al., 2015). In addition, the local administration of drugs in the peritoneal or pleural space might be an improved strategy to treat MM. Towards this end, several clinical trials have been approved for intrapleural delivery of targeted therapy in pleural MM (ClinicalTrials.gov, 2017). Intra serous sac therapies are hypothesized to have better antitumor activity because the agent would be in direct contact with cancer cells and it could reach higher concentrations by limiting the side effects. The majority of intracavitary chemotherapy trials have used platinum-based regimens, but despite the local administration of the drug side effects occurred (Chang and Sugarbaker, 2004). Intrapleural gene therapy and immunotherapy have been also employed but these therapies appear to be more advantageous in patients with small tumors (Gomez and Tsao, 2014). The combination of targeted therapies has shown to increase anti-tumor activities (Favoni and Florio, 2011). A “multifunctional drug” is a molecule able to modulate the activity of multiple targets involved in carcinogenesis through direct interaction or modulation of gene expression (Masuelli et al., 2014). Polyphenols are considered multifunctional drugs (Benvenuto et al., 2013; Fantini et al., 2015; Stedile et al., 2016). We recently demonstrated that the intraperitoneal administration of curcumin reduced the growth of MM #40a cells transplanted in the peritoneal cavity of C57BL/6 mice (Masuelli et al., 2017). Among the others the polyphenol apigenin was shown to have anti-tumoral *in vitro* activity by targeting multiple signaling pathways (Lepley and Pelling, 1997; Yin et al., 2001; Ujiki et al., 2006; Shukla and Gupta, 2007; Hussain et al., 2010; Shukla and Gupta, 2010; Masuelli et al., 2011; Bao et al., 2013; Tong and Pelling, 2013; Chen et al., 2014; Lee et al., 2014; Wu et al., 2014; Liu et al., 2015; Seo et al., 2015; Shi et al., 2015; Shukla et al., 2015; Kim et al., 2016; Lim et al., 2016; Sung et al., 2016; Xu et al., 2016; Ganai, 2017).

Here, we demonstrated for the first time that API treatment was able to inhibit the growth of MM cell lines *in vivo*. Indeed, the intraperitoneal administration of API reduced the growth of MM #40a cells transplanted in the peritoneal of C57BL/6 mice. The risk of developing tumors in vehicle treated mice was 23.17 in comparison to those treated with 20 mg/kg API one time per week. Only few data are available on the *in vivo* antitumor activity of API. Budhraja et al. reported the attenuation of tumor growth in U937 xenografts by API (Budhraja et al., 2012). Shukla and Gupta showed the induction of p53-mediated apoptosis in prostate cancer by *in vivo* API administration (Shukla and Gupta, 2008). In addition, API has been demonstrated to synergize with chemotherapeutic agent such as gemcitabine and 5-fluorouracil *in vivo* (Lee et al., 2008; Hu et al., 2015). API has been demonstrated to modulate immune response by inhibiting regulatory T cells (Tregs) and increasing CD4+ and CD8+ T cells at the tumor site in pancreatic cancer (Nelson et al., 2017). The presence of Tregs has been demonstrated in MM microenvironment (Ying et al., 2015). Thus, the use of a drug that is able to simultaneously modulate immune response and inhibit cancer cells growth might represent a promising tool for MM treatment.

Apigenin is a potent pleiotropic molecule being able to simultaneously inhibit different signaling transduction pathways (Sung et al., 2016). Thus, the effects of API were also analyzed *in vitro* employing human and mouse MM cells. Here we demonstrate that API induced apoptosis in MM cells. Recent reports suggest a role for ROS in inducing apoptosis by cells treatment with several drugs, including chemopreventive agents (Singh et al., 2005). We demonstrated that API induced ROS generation resulting in DNA damage and up-regulation of p53. One of the most characterized mechanisms of API is the ability to induce apoptosis through the p53-related pathway (Sung et al., 2016). In addition, it has been demonstrated that API was able to increase the production of ROS which induced up-regulation of p53 in prostate cancer and in hepatoma cancer cells (Shukla and Gupta, 2008; Zhang et al., 2015). Accordingly, we demonstrated that the increase of p53 expression was paralleled by the up-regulation of Bax protein expression, the increase in the Bax/Bcl-2 ratio and the activation of caspase 9 in API-treated MM cells.

Apigenin is a histone deacetylases (HDAC) inhibitor. HDACs are emerging as remarkable molecular targets for anticancer drugs and therapy. HDAC inhibitors (HDACi) induce apoptosis limiting the function of HDACs, thus declining the levels of antiapoptotic proteins (Black and Morris, 2012; Ganai, 2017). Several clinical trials using HDACi alone or in combination with chemotherapy have been approved for treatment of human MM. Moreover, HDACi have been demonstrated to sensitize malignant cells to EGFR inhibition in non-small cell lung carcinoma (Lindemann et al., 2007). We previously demonstrated that API was able to selectively inhibit the tyrosine phosphorylation of specific residues of EGFR and ErbB2 in head and neck squamous cell carcinoma (Masuelli et al., 2011). Here, we demonstrated that API treatment decreases the expression of both EGFR and ErbB2. This might be due to API-induced proteasomal degradation through polyubiquitination as demonstrated for ErbB2/neu (Pandey et al., 2012). Thus, the use of API can mimic the effects of HDAC inhibitors and EGFR/ErbB2-TKI in a unique molecule.

In addition, we report that API treatment activates the extrinsic apoptotic pathway by up-regulating the levels of cleaved caspase-8 (Liao et al., 2014).

Autophagy can act either as a tumor-suppressor or as a tumor-promoter (Zhan et al., 2016). Clinical trials are performed using autophagy inhibitors in combination with other drugs to treat different types of cancer (Towers and Thorburn, 2016). We recently demonstrated that treatment with curcumin in MM cell lines was able to trigger the autophagic flux but that the process was then blocked to promote MM cells apoptosis (Masuelli et al., 2017). It has been described that API is able to activate autophagy in several human cancer cell lines (Sung et al., 2016). The increased levels of ROS and DNA damage can activate autophagy via activation of different signal transduction pathways. However, treatment with API did not trigger autophagy in our model, despite the inhibition of AKT phosphorylation. API has been demonstrated to modulate directly or indirectly several signal transduction pathways including PI3K/AKT and NF-κB (Ganai, 2017). Recent studies have reported that the effects of API-mediated down-regulation of the PI3K/AKT pathways is dependent on the inhibition of GLUT-1 expression (Bao et al., 2013). Several studies have shown that AKT regulates the NF-κB pathway via phosphorylation and activation of molecules of the NF-κB signaling pathway. Inhibition of activation of IKKα led to a decrease in the phosphorylation status of IκBα that in its unphosphorylated form maintains NF-κB blocked in the cytoplasm (Romashkova and Makarov, 1999; Sarkar and Li, 2004). We demonstrated that API treatment was able to inhibit nuclear translocation of NF-κB in all human MM cell lines. This event was paralleled by the decrease of phosphorylation and/or expression of IκBα, probably due to inhibition of AKT activation. In addition, the inhibition of NF-κB activity could induce apoptosis by down-modulating the expression of its regulated genes including Bcl-2. Overall, API-mediated-inhibition of NF-κB, and AKT signaling might inhibit pro-survival signals inducing apoptosis in MM cells.

The effect of API on MAPKs-mediated signaling pathways remains unclear. Several studies have reported increase or decrease phosphorylation of MAPKs pathways, depending on cell type (Kim et al., 2013; Sui et al., 2014; Wu et al., 2015; Lim et al., 2016). No data are available on the effects of apigenin on ERK1/2 activation in MM cell lines. Here, we demonstrated that API treatment was able to increase ERK1/2 phosphorylation in MM-B1, H-Meso-1, and #40a cells while induced a decrease of ERK1/2 activation in MM-F1 cells. The opposite effect might due to different histotypes of the MM cell lines reflecting the natural tumor heterogeneity occurring in humans. Moreover, API treatment induced the activation of p38 MAPK in all MM cells except in H-Meso-1 cells, and the inhibition of JNK p54 in all MM cells with the exception of MM-F1 cells. A decrease in JNK p46 was also demonstrated in MM-B1 and #40a cell lines. API has been demonstrated to activate or inhibit both p38 and JNK pathways depending on cell type (Wu et al., 2015; Sung et al., 2016). The p38 MAPK is strongly activated by oxidative and genotoxic stresses and regulates apoptosis, cell cycle arrest and growth inhibition (Sui et al., 2014). c-Jun N-terminal kinase (JNK) is also activated in response to a variety of stress signals and has been implicated in regulation of apoptosis and autophagy (Sui et al., 2014). The activated JNK regulates a variety of transcription factors such as c-Jun and c-Fos. Thus, API-dependent activation of p38 could mediate the activation of apoptosis. API-dependent inhibition of JNK by decreasing the phosphorylation and expression of c-Jun in MM cells resulted in the inhibition of MM cell growth.

## Conclusion

Overall, we demonstrated that Apigenin inhibited *in vitro* and *in vivo* malignant mesothelioma cells growth by targeting different signaling pathways and inducing apoptosis. Our findings may have important implications for the design of MM treatment using API in addition to other drugs.

## Author Contributions

LM performed Western blotting analysis, analyzed the results, and wrote the manuscript. MB performed FACS analysis, statistical analysis. RM performed cell death experiments and *in vivo* experiments. EDS performed Western blotting analysis and ROS production. EZ and GB performed Western blotting and immunofluorescence analyses. GT performed cell proliferation and *in vivo* experiments. IT, MG, GF, AM critically revised the manuscript. RB supervised the project, analyzed the results, and wrote the manuscript.

## Conflict of Interest Statement

The authors declare that the research was conducted in the absence of any commercial or financial relationships that could be construed as a potential conflict of interest.
